# Uropathogenic *Escherichia coli* Superinfection Enhances the Severity of Mouse Bladder Infection

**DOI:** 10.1371/journal.ppat.1004599

**Published:** 2015-01-08

**Authors:** Drew J. Schwartz, Matt S. Conover, Thomas J. Hannan, Scott J. Hultgren

**Affiliations:** 1 Department of Molecular Microbiology, Center for Women's Infectious Disease Research, Washington University in St. Louis, St. Louis, Missouri, United States of America; 2 Department of Pathology & Immunology, Washington University in St. Louis, St. Louis, Missouri, United States of America; Stanford University School of Medicine, United States of America

## Abstract

Urinary tract infections (UTIs) afflict over 9 million women in America every year, often necessitating long-term prophylactic antibiotics. One risk factor for UTI is frequent sexual intercourse, which dramatically increases the risk of UTI. The mechanism behind this increased risk is unknown; however, bacteriuria increases immediately after sexual intercourse episodes, suggesting that physical manipulation introduces periurethral flora into the urinary tract. In this paper, we investigated whether superinfection (repeat introduction of bacteria) resulted in increased risk of severe UTI, manifesting as persistent bacteriuria, high titer bladder bacterial burdens and chronic inflammation, an outcome referred to as chronic cystitis. Chronic cystitis represents unchecked luminal bacterial replication and is defined histologically by urothelial hyperplasia and submucosal lymphoid aggregates, a histological pattern similar to that seen in humans suffering chronic UTI. C57BL/6J mice are resistant to chronic cystitis after a single infection; however, they developed persistent bacteriuria and chronic cystitis when superinfected 24 hours apart. Elevated levels of interleukin-6 (IL-6), keratinocyte cytokine (KC/CXCL1), and granulocyte colony-stimulating factor (G-CSF) in the serum of C57BL/6J mice prior to the second infection predicted the development of chronic cystitis. These same cytokines have been found to precede chronic cystitis in singly infected C3H/HeN mice. Furthermore, inoculating C3H/HeN mice twice within a six-hour period doubled the proportion of mice that developed chronic cystitis. Intracellular bacterial replication, regulated hemolysin (HlyA) expression, and caspase 1/11 activation were essential for this increase. Microarrays conducted at four weeks post inoculation in both mouse strains revealed upregulation of IL-1 and antimicrobial peptides during chronic cystitis. These data suggest a mechanism by which caspase-1/11 activation and IL-1 secretion could predispose certain women to recurrent UTI after frequent intercourse, a predisposition predictable by several serum biomarkers in two murine models.

## Author Summary

Urinary tract infections (UTIs) affect millions of women each year resulting in substantial morbidity and lost wages. Approximately 1.5 million women are referred to urology clinics suffering from chronic recurrent UTI on a yearly basis necessitating the use of prophylactic antibiotics. Frequent and recent sexual intercourse correlates with the development of UTI, a phenomenon referred to clinically as “honeymoon cystitis.” Here, using superinfection mouse models, we identified bacterial and host factors that influence the likelihood of developing chronic UTI. We discovered that superinfection leads to a higher rate of chronic UTI, which depended on bacterial replication within bladder cells combined with an immune response including inflammasome activation and cytokine release. These data suggest that bacterial inoculation into an acutely inflamed urinary tract is more likely to lead to severe UTI than bacterial presence in the absence of inflammation. Modification of these risk factors could lead to new therapeutics that prevent the development of recurrent UTI.

## Introduction

Nearly nine million people present each year to primary care physicians with a urinary tract infection (UTI), costing nearly $2 billion yearly [1], [2]. Women suffer the majority of these infections, with the lifetime risk approaching 50% [3]. Furthermore, 25–40% of these women will suffer recurrent UTI (rUTI), with 1.5 million women referred to urology clinics and often requiring prophylactic antibiotics to prevent recurrence [4]–[6]. Uropathogenic *E. coli* (UPEC) are responsible for>80% of community acquired UTI and 50% of nosocomial UTI [7], [8]. In the absence of antibiotic therapy, up to 60% of women experience symptoms and/or bacteriuria lasting months after initial infection [9]–[12], implying that cystitis is not always self-limiting. Furthermore, if the infection persists without adequate treatment, the organisms have the capacity to ascend the ureters, causing pyelonephritis and sepsis [13]. Antibiotic resistant organisms further complicate infection and threaten to increase the likelihood of chronic UTI, pyelonephritis and potentially bacteremia [14], [15]. UTIs are increasingly being treated with fluoroquinolones, which in turn has led to a rise in resistance and the spread of multi-drug resistant microorganisms globally, which is a looming worldwide crisis [16], [17]. It is therefore imperative to understand the molecular mechanisms that underlie this problematic disease in order to develop novel therapies.

Sexual intercourse is one of the most significant risk factors predisposing otherwise healthy women to UTI. Early studies demonstrated that sexual intercourse led to a 10-fold increase in bacteria/ml of urine and a subsequently increased predisposition to developing a UTI within 24 hours thereafter [5], [18]–[21]. More recent studies have shown that the frequency with which a woman has sexual intercourse dramatically impacts the likelihood of developing both acute and rUTI [4], [22], [23]. Scholes *et. al* found a direct association between the number of episodes of sexual intercourse in a given month and the risk of developing rUTI. However the significance of the timing between these episodes of sexual intercourse is unknown. Are evenly spaced episodes associated with an equal risk or, instead, does an episode prime the bladder for rUTI if another insult follows within a sensitive period? To address this question, we developed a model of sequential infection in mice to explore the hypothesis that a sensitive period exists after an initial bacterial insult to the bladder in which the likelihood of developing severe, chronic infection is dramatically increased.

Murine models of UTI have been used to decipher complexities of this disease in naïve individuals. UPEC are capable of colonizing multiple body habitats and niches, including both intracellular and extracellular locations within the bladder, as well as in the gastrointestinal (GI) tract and the kidneys. Selective pressure and bacterial population bottlenecks during colonization impact the ultimate fate of disease [24]–[27]. Adhesive pili assembled by the chaperone/usher pathway (CUP), such as type 1 pili, contain adhesins at their tips that function in adherence and invasion of host tissues and in biofilm formation on medical devices. Upon introduction of UPEC into the bladder, bacteria bind to either mannosylated uroplakin plaques or β1-α3 integrin receptors on the epithelial surface of the bladder via the type 1 pilus FimH adhesin [28]–[30]. Upon internalization, UPEC can be exocytosed as part of a TLR4 dependent innate defense process [31]. In addition to expulsion of individual bacteria, the host can exfoliate superficial facet cells to shed attached and invaded bacteria into the urine for clearance [29]. A small fraction of invaded bacteria escape into the host cell cytoplasm, where they are able to subvert expulsion and innate defenses by replicating into biofilm-like intracellular bacterial communities (IBCs) [24], [32]. UPEC eventually flux out of these communities with a substantial proportion existing as neutrophil resistant filaments [33], [34]. Importantly, evidence of IBCs and bacterial filaments have been observed in women suffering acute UTI, one to two days post self-reported sexual intercourse, but not in healthy controls or infections caused by Gram-positive organisms, which do not form IBCs [21]. IBCs have also been observed in urine from children with an acute UTI [35]. Additionally, IBC formation and the innate immune response of cytokine secretion and exfoliation have been observed in all tested mouse strains, but the long-term outcome of infection differs [36]–[38].

There are two main, mutually exclusive, outcomes to acute infection in C3H/HeN mice: either chronic bacterial cystitis (chronic cystitis), which is characterized by persistent high titer bacteriuria (>10^4^ CFU/ml) and high titer bacterial bladder burdens (>10^4^ CFU) two or more weeks after inoculation, accompanied by chronic inflammation [37], [39], or resolution of bacteriuria [37]. Mice that resolve infection may harbor small populations of dormant UPEC called Quiescent Intracellular Reservoirs (QIRs) [40]. Other mouse strains exhibit varied proportions of these two outcomes. C57BL/6J mice resolve bacteriuria within days and thus are resistant to chronic cystitis, but are susceptible to QIR formation [40], [41]. In contrast, other TLR4-responsive C3H background sub-strains and closely related CBA/J and DBA/2J mice experience persistent high-titer bacteriuria and bladder colonization by UPEC in the presence of chronic inflammation lasting at least four weeks post-infection (wpi). During chronic cystitis, persistent lymphoid aggregates and urothelial hyperplasia with lack of superficial facet cell terminal differentiation accompany luminal bacterial replication [37]. These same histological findings of submucosal lymphoid aggregates and urothelial hyperplasia have been observed in humans suffering persistent bacteriuria and chronic cystitis [42]. Since murine chronic cystitis predisposes to recurrent chronic UTI after antibiotic-mediated bacterial clearance, this is also a relevant model to interrogate the mechanism of recurrent cystitis [37]. In mouse models of UTI, mice initially experience urinary frequency and dysuria as determined by reaction to noxious stimuli and nerve responses during acute infection [43], [44]; however, during chronic cystitis bacterial replication may exist in an asymptomatic carrier state as studies have not been conducted to determine whether dysuria persists. Interestingly, higher serum levels of interleukins (IL) 5 and 6, keratinocyte cytokine (KC/CXCL1), and granulocyte colony-stimulating factor (G-CSF) in C3H/HeN mice at 24 hours post infection (hpi) predicted the development of persistent bacteriuria and chronic cystitis thereafter, suggestive of a host-pathogen checkpoint during acute infection that predicts long term outcome [26], [37]. In women with an acute UTI, increased amounts of serum CXCL1, M-CSF, and IL-8 correlated with subsequent rUTI, suggesting a similar checkpoint [45].

In this manuscript, we developed a superinfection model to mimic the clinical scenario of frequent sexual intercourse whereby sequential inocula are introduced within a brief period of time. C57BL/6J mice are resistant to chronic cystitis when singly infected; however, 30% of C57BL/6J mice developed chronic cystitis when superinfected 24 hours after the initial infection. Serum elevations of IL-6, KC, and G-CSF prior to superinfection predicted the development of persistent bacteriuria in C57BL/6J mice similar to singly infected C3H/HeN mice. Superinfecting C3H/HeN mice 1–6 hours after the initial inoculation increased the proportion of mice experiencing chronic cystitis. In order for this elevation to occur, we found that the initial UPEC inoculum (the “priming” inoculation) must be alive, invasive, capable of intracellular replication, and able to regulate hemolysin expression. Inhibition of the caspase 1/11 inflammasome prior to priming reduced bacterial CFU at four wpi relative to DMSO-treated mice. Microarray analysis of mouse bladders four wpi revealed that both C57BL/6J and C3H/HeN mice secreted antimicrobial peptides and IL-1 during chronic infection. In contrast to C3H/HeN mice, immunoglobulin expression was upregulated in C57BL/6J mice experiencing chronic cystitis. This immunoglobulin expression was absent in C57BL/6J mice that resolved infection and in C3H/HeN mice. Our data suggest mechanisms whereby certain women may be susceptible to rUTI after frequent sexual intercourse dependent on intracellular bacterial replication and the host immune response.

## Results

### Time-sensitive enhancement of infection

**Figure 1:**
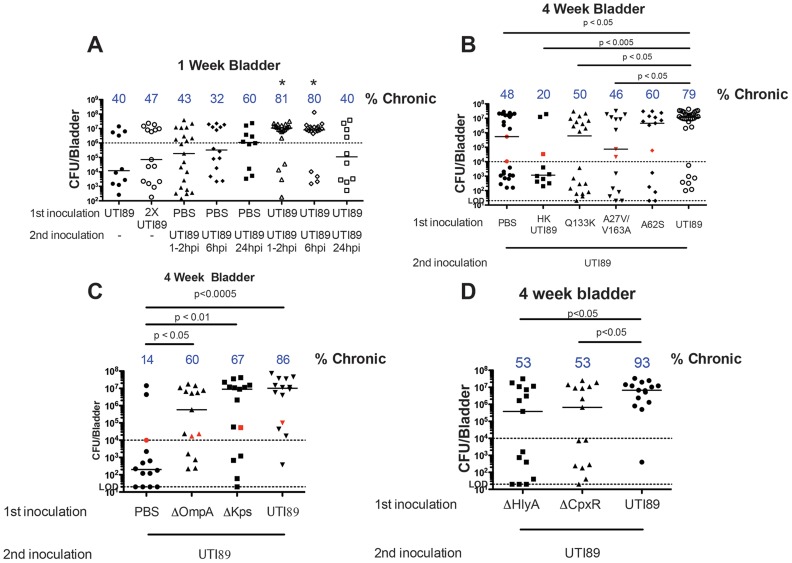
Superinfections of C3H/HeN mice. **A**) Mice were infected with 10^7^ CFU UTI89, 2×10^7^ CFU UTI89, or PBS and re-infected with UTI89 at the indicated time points. One week total bladder titers are shown. Percentage of mice likely to develop chronic cystitis is displayed at the top of the column based on a CFU cutoff of 10^6^. Asterisks indicate p<0.05 from the PBS control and singly infected mice. **B–D**) Mice were infected with the indicated strain or PBS and re-infected one hour later. Urine titers were determined over time and four-week bladder titers are displayed. The fraction of mice with chronic cystitis is displayed at the top of each column. Red data points indicate resolved infection. Horizontal bars indicate median values. The dashed line at 20 CFU represents the LOD, and the dashed line at 10^6^ (**A**) or 10^4^ CFU (**B–D**) represents the chronic cystitis cutoff for urine and bladder titers. Panel A reflects 2–4 experiments with 5–9 mice per group. Panel B is 2–7 experiments with 4–5 mice per group. Panel C–D are 3 experiments with 4–5 mice per group. Statistical comparisons were determined using Fisher's exact test based on the fraction of mice experiencing chronic cystitis.

Studies suggest that a host-pathogen checkpoint within the first 24 hpi determines UTI outcome in C3H/HeN mice [26], [37]. In addition, the chronic inflammation observed in mice experiencing chronic cystitis was found to predispose to rUTI after re-infection [37]. Thus, we hypothesized that superinfecting mice during this period of acute inflammation would increase the proportion of mice experiencing chronic cystitis. We transurethrally infected 7–8 week old female C3H/HeN mice with 10^7^ CFU UTI89 or PBS as the priming inoculation and superinfected them 1–2, 6, or 24 hours thereafter. Enumeration of bacterial CFU at one wpi as an initial screen revealed a dramatic increase in the proportion of mice experiencing chronic cystitis in mice superinfected 1–6 hours after priming compared to singly infected or PBS treated mice (Fig. 1A). We used a cutoff of 10^6^ CFU to demarcate mice experiencing high-titer bacterial infection at one week. Importantly, we did not observe a significant increase in CFU when a single inoculum was doubled (2×10^7^ CFU). Superinfection at 24 hpi had no effect on bacterial titers at one week, suggesting that the factors predisposing to increased susceptibility to chronic cystitis upon superinfection wane over time [26]. However, inoculation with PBS followed by UTI89 24 hpi did lead to high titers in 60% of mice. While this result is perplexing, it possibly reflects that sacrifice six days post infection was not sufficient to delineate the typical bimodal distribution of outcomes [37]. The process of catheterization also induces inflammation, which may not have resolved by 6 dpi [46]. We conducted all subsequent C3H/HeN superinfections one hour after priming.

Since early severe inflammatory responses predispose to chronic cystitis [37], we hypothesized that the initial inoculum primed the bladder by initiating an innate immune response to intracellular bacteria that predisposed to a higher proportion of mice experiencing chronic cystitis upon superinfection. We utilized a panel of UTI89 mutants in *fimH*, *ompA*, and *kps* that have been shown to differ in their ability to: i) invade and form IBCs and ii) persist during chronic cystitis in co-infection experiments [47], [48]. Mature IBCs caused by WT bacteria are clonally derived from a single invasive event [24]. The mannose-binding pocket of FimH is invariant among sequenced UPEC [47], and the binding pocket mutant, FimH::Q133K, is defective in mannose-binding and can neither invade the bladder epithelium nor form IBCs. FimH undergoes compact and elongated conformational changes wherein the receptor binding domain bends approximately 37° with respect to the pilin domain. The mannose-binding pocket is deformed in the compact conformation whereas the elongated conformation is mannose binding proficient [49], [50]. Several residues outside the mannose-binding pocket (positions 27, 62, 66 and 163) are under positive selection in clinical UPEC isolates compared to fecal strains [47] and have been shown to function in modulating the conformational changes between the elongated and compact states [48]. FimH::A27V/V163A predominantly adopts a high-mannose binding, elongated conformation. Its expression results in: i) a 10-fold reduction in intracellular CFU one hpi and ii) a defect in the ability to form IBCs at six hpi. FimH::A62S shifts the equilibrium towards the compact conformation. Expression of this allele results in: i) a 10-fold reduction in intracellular CFU one hpi and ii) a 10-fold reduction in IBC formation compared to WT UTI89 [47], [48]. UTI89Δ*ompA* forms half the number of IBCs as UTI89 [51], and UTI89Δ*kps* is defective in IBC formation. UTI89Δ*kps* can replicate intracellularly and the IBC defect can be rescued by co-inoculation with WT UTI89, which results in mixed strain, non-clonal, IBCs [52].

We primed mice with these strains and superinfected one hpi with WT UTI89 and assessed bacteriuria at days 1, 7, 14, and 21 and enumerated bladder titers at 28 dpi. Mice were designated as having chronic cystitis if they had urine bacterial titers greater than 10^4^ CFU/ml at each time point *and* bladder titers greater than 10^4^ CFU at sacrifice [37]. We found that the FimH::A27V/V163A allele was incapable of priming the bladder for the development of chronic cystitis (p<0.05 relative to WT superinfection). In contrast, FimH::A62S did not significantly differ from PBS or WT superinfection; therefore, it may be capable of priming, though to a lesser degree. UTI89Δ*ompA* and UTI89Δ*kps* were both able to prime the bladder for enhanced chronic cystitis relative to PBS when superinfected one hpi with WT UTI89 (p<0.05 and p<0.01 respectively; Fig. 1C). We also primed with heat-killed UTI89 and found that live, but not heat killed, UTI89 were capable of priming the bladder indicating that bacterial products such as LPS were insufficient (Fig. 1B). These data indicate that live and invasive UTI89 capable of at least some degree of intracellular replication are required for the priming to enhance the incidence of chronic cystitis upon superinfection of UTI89. Taken together these data suggest that priming begins during invasion and early IBC formation.

### UPEC hemolysin and caspase 1/11 activation are essential

One of the most potent host defenses to eliminate adherent and invaded UPEC is superficial facet cell exfoliation [29]. The process of exfoliation is activated in part by the bacterial expression of hemolysin (HlyA) [53](Nagamatsu *et al.* in review). UTI89Δ*cpxR* overexpresses HlyA, leading to exfoliation and attenuation in our murine model of cystitis (Nagamatsu *et al.* in review). The UTI89Δ*cpxR*Δ*hlyA* double mutant was not attenuated, suggesting that the *in vivo* defect was due to increased hemolysin expression (Nagamatsu *et al.* in review). The ability of UPEC to rapidly build up in numbers in the form of IBCs and then disperse to neighboring cells may be part of a mechanism to subvert an exfoliation response. Thus, fine-tuning the expression of HlyA during acute bladder infection may serve to maximize UPEC persistence and give UPEC a fitness edge against the host innate inflammatory response. Interestingly, in C3H/HeN mice, UTI89 Δ*hlyA* is not attenuated throughout infection and causes chronic cystitis comparable to UTI89; however, other reports suggest deletion of HlyA in UPEC CFT073 decreases virulence [54]. We investigated the role of hemolysin in priming the bladder for chronic cystitis upon superinfection by utilizing UTI89Δ*hlyA* or UTI89Δ*cpxR* as the initial inoculation followed by WT UTI89 one hpi. Both of these strains were statistically significantly different when compared to WT UTI89 as the priming inoculum. Therefore, we conclude that neither was capable of priming the bladder for enhanced chronic cystitis (Fig. 1D). Thus, too high or low expression of hemolysin abolished the ability of UTI89 to prime for enhanced chronic cystitis implying that an optimal level of hemolysin expression is critical for priming the bladder for enhanced chronic cystitis.

**Figure 2:**
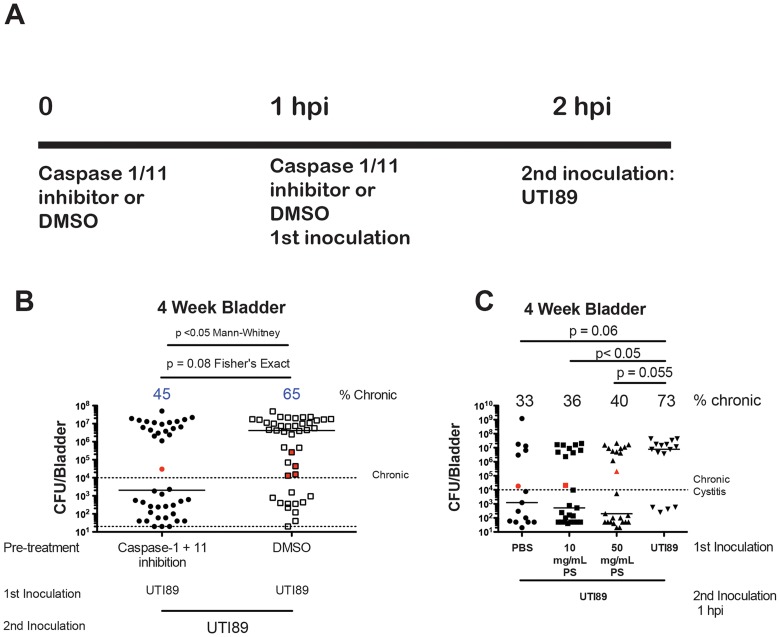
Role of caspase 1/11 and exfoliation in C3H/HeN superinfections. **A**) Inoculation protocol shown for caspase inhibition studies of panel B. **B**) Four-week total bladder titer based on inhibitor or vehicle. **C**) Mice were inoculated with PBS, UTI89 or the indicated dose of protamine sulfate in 50 µL PBS and inoculated one hour later with UTI89. Urine was collected weekly and overall bladder titers are shown at four weeks. Panel **B** represents 5 experiments with n = 5–10 mice per group. Panel **C** represents 2 experiments with n = 5–10 mice per group. **B–C**) Observations in red indicate resolved infection. Percent of mice with persistent bacteriuria and chronic cystitis is shown at the top of each column. For Panel **B** and **C**, a Fisher's Exact Test was used to determine significance between proportions of mice experiencing chronic cystitis. Mann-Whitney U Test was used to compare median CFU values in Panel **B**.

HlyA-mediated exfoliation is in part due to its ability to trigger degradation of paxillin, a scaffold protein that modulates the dynamics of cytoskeletal rearrangements [55]. HlyA can also trigger cell death in human bladder epithelial cells and release of IL-1α via caspase-4 (the murine ortholog is caspase-11) activation and caspase-1-dependent IL-1β secretion via activation of the NLRP3 inflammasome pathway, which orchestrates additional cell death (Nagamatsu *et al.* in review). We hypothesized that inflammasome and caspase 1/11 activation were essential for superinfection. Thus, mice were treated intravesically with a dose of caspase 1/11 inhibitor or DMSO one hour prior to priming and a second dose with the priming inoculum to test this hypothesis (Fig. 2A). Providing two doses of the inhibitor was previously shown to be effective in dampening *in vivo* inflammatory responses. *In vitro*, the inhibitor dramatically reduced downstream elements of inflammasome activation, IL-1α and IL-1β secretion, when bladder cells were infected with UTI89 (Nagamatsu *et al.* in review). Caspase 1/11 inhibition significantly reduced median bladder titers at four weeks after superinfection relative to the DMSO control group (Fig. 2B). We also saw a trend of caspase 1/11 inhibition in reducing the proportion of WT superinfected mice experiencing chronic cystitis to single infection levels (Fig. 2B). DMSO also reduced the proportion of mice experiencing persistent bacteriuria and chronic cystitis, but to a lesser degree than caspase 1/11 inhibition (Fig. 2B vs. Fig. 1B–D), suggesting an anti-inflammatory role of DMSO alone. Intriguingly, DMSO was recently found to inhibit the NLRP3 inflammasome [56]. Taken together, these data implicate hemolysin and the NLRP3 inflammasome in the priming response to enhanced chronic cystitis.

We further investigated whether chemical exfoliation could enhance the proportion of mice experiencing chronic cystitis prior to a single infection. We utilized the cationic protein, protamine sulfate, which has previously been used to exfoliate the superficial facet cell layer of the urothelium [40], [57]. A 10 mg/mL dose delivered intravesically in 50 µL PBS was shown to exfoliate 65% of the facet cell layer 12 hours after treatment while an additional booster dose of 50 mg/mL led to 95% exfoliation [40]. We utilized these concentrations to initiate, but likely not complete, the process of exfoliation one hour prior to infection with UTI89. We did not observe a significant increase in the proportion of mice experiencing chronic cystitis over PBS pretreatment (Fig. 2C). Thus, these data suggest that at least partial IBC formation in conjunction with caspase 1/11 activation primes the bladder for enhanced chronic cystitis, but chemical initiation of exfoliation is not sufficient. Taken together, these data suggest that exfoliation per se might not play a significant role in impacting the likelihood of enhanced chronic cystitis but instead may reflect a downstream marker of the priming event.

### Superinfection leads to chronic cystitis in a resistant mouse strain

**Figure 3:**
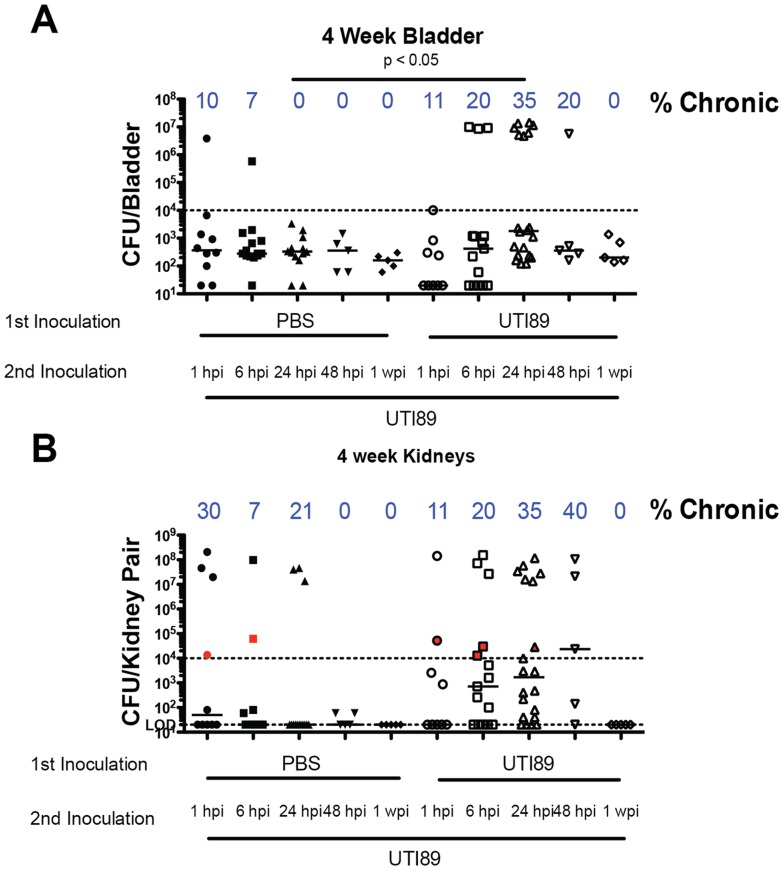
C57BL/6J mice are susceptible to chronic cystitis when superinfected 24 hpi. **A–B**) Mice were transurethrally infected with PBS or UTI89 and re-infected at the indicated timepoints with UTI89. Urine was tracked weekly and four-week total bladder (**A**) and kidney pair (**B**) titer is displayed. N = 2–4 experiments with 4–5 mice per group. Statistical differences determined by Fisher's Exact test.

**Figure 4:**
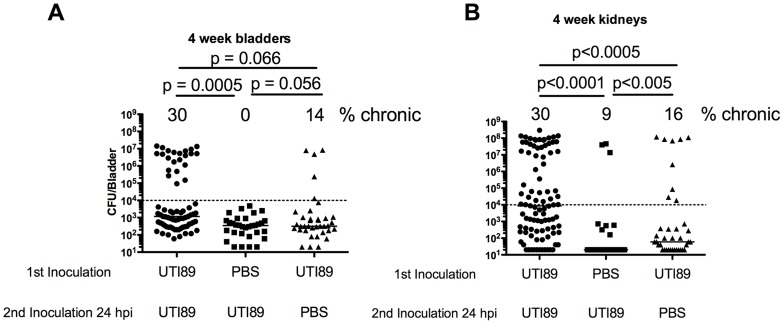
UTI89 Superinfection of C57BL/6J mice increases bladder and kidney infection. **A–B**) mice were infected with PBS or UTI89 Kan^r^ and re-infected 24 hrs later with PBS or UTI89 Spect^r^. Urine was tracked over four weeks and total bladder (**A**) and kidney pair titer (**B**) is displayed. Number above columns indicates number of mice with persistent bacteriuria with bladder (**A**) or kidney (**B**) titer>10^4^ CFU. Data represents 3–8 experiments with n = 4–29 mice per group. Panels also include data reproduced from Fig. 3. Statistical differences determined by Fisher's Exact test.

C57BL/6J mice typically rapidly resolve bacteriuria and are resistant to chronic cystitis upon single inoculation with UPEC [37], [38]. Five to ten percent of the time after inoculation with UTI89, C57BL/6J mice experience persistent bacteriuria, but this is generally due to kidney infection without concomitant high titer bladder infection [37], [41]. This degree of kidney infection is not infectious dose dependent and therefore likely due to ureteric reflux of the bacteria during experimental inoculation [37]. We investigated whether superinfecting C57BL/6J mice during acute infection would stimulate an immune response leading to chronic cystitis. We inoculated bladders with PBS or 10^7^ CFU of UTI89 followed by superinfection with UTI89 1, 6, 24, 48 hours or one week after initial infection and collected urine at days 1, 7, 14, and 21 dpi followed by enumeration of bladder and kidney titers at 28 dpi (Fig. 3). A 24 hpi superinfection resulted in 35% of mice sustaining persistent bacteriuria with bladder titers >10^4^ CFU at four weeks compared to 0% in the singly infected group (Fig. 3A). Kidney titers were also increased in the mice with persistent bacteriuria, but we did not observe a significant increase in the proportion of mice with kidney infection greater than 10^4^ CFU (Fig. 3B). These data suggest that at 24 hours after infection the bladders of C57BL/6J mice were primed to develop chronic cystitis upon superinfection. We investigated whether an ascending kidney infection plays a role in predisposing these mice to chronic cystitis by inoculating PBS into the bladder, either 24 hours before or after infection with UTI89, to stimulate a bladder and ureter stretch response or potentially increase reflux of bacteria into the kidneys, respectively. We determined the percentage of mice with persistent bacteriuria and those with bladder and kidney titers greater than 10^4^ CFU at sacrifice (Table 1). We found in all conditions that persistent bacteriuria was a 100% predictor of kidney titers>10^4^ CFU at four wpi. Persistent bacteriuria also predicted bladder titers greater than 10^4^ CFU at four wpi in C57BL/6J mice superinfected 24 hpi with UTI89. For the group of mice inoculated with PBS before the initial UTI89 infection, persistent bacteriuria did not correlate with high bladder titers suggesting these bacteria were only replicating in the kidneys. Serially infecting with two inocula of UTI89 trended towards increased persistent bacteriuria and chronic cystitis compared to the group inoculated with UTI89 followed by PBS at 24 hpi (P = 0.066; Table 1 and Fig. 4A). Kidney titers of UTI89 superinfected mice were significantly higher than when PBS was used to prime or superinfect perhaps suggesting that repeat infection may also increase susceptibility to pyelonephritis (Fig. 4B). Thus, a 24 hpi superinfection of WT UTI89 led to increased rates of persistent bacteriuria and chronic cystitis; however, bladder/ureter stretch or kidney ascension at 24 hpi may contribute to this increase.

**Table 1:** Characteristics of C57BL/6J superinfections.

Condition^a^	Incidence % (n)	Persistent Bacteriuria^b^		Resolved Bacteriuria		Persistent Bacteriuria		Resolved Bacteriuria	
		Bladder Titers^c^^,^ d	PPV^e^	Bladder Titers	NPV^e^	Kidney Titers	PPV^e^	Kidney Titers	NPV^e^
UTI89→UTI89	30 (28/94)	4.4×10^6^	92 (22/24)	5.5×10^2^	100 (50/50)	3.6×10^7^	100 (28/28)	1.2×10^3^	73 (49/67)
UTI89→PBS	16 (6/37)	2.5×10^6^	83 (5/6)	3.2×10^2^	100 (31/31)	8.6×10^7^	100 (6/6)	6.0×10^1^	90 (28/31)
PBS→UTI89	9 (3/32)	1.9×10^3^	0 (0/3)	3.4×10^2^	100 (29/29)	4.0×10^7^	100 (3/3)	2.0×10^1^	100 (29/29)

a All mice infected 24 hours after initial infection.

b Defined as>10^4^ CFU/mL bacteria in clean catch urine throughout four-week infection.

c Median values listed.

d Bladders of 20 mice were used for imaging purposes so no titers were available.

e PPV is the positive predictive value of persistent bacteriuria predicting bladder/kidney titer greater than 10^4^ CFU. NPV is the negative predictive value of resolved bacteriuria predicting bladder/kidney titer less than 10^4^ CFU.

**Figure 5:**
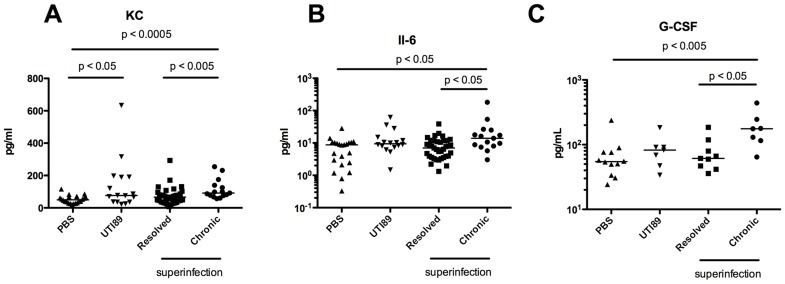
Serum cytokine signature of C57BL/6J mice with persistent bacteriuria. Serum was obtained 24 hrs after initial inoculation prior to superinfection. Levels of 23 cytokines were determined and cytokines showing significant differences between resolved and chronic superinfected mice are shown. Levels of KC (**A**), IL-6 (**B**), and G-CSF (**C**) shown in pg/mL. Data represent 4–6 experiments with n = 4–29 mice per group. Statistical differences determined by One-Way ANOVA overall and Mann-Whitney U test for pairwise comparisons.

C3H/HeN mice that progress to chronic cystitis upon single inoculation can be predicted by elevated serum levels of IL-5, IL-6, KC, and G-CSF at 24 hpi [37]. We hypothesized that similar elevations would predict sensitization to chronic cystitis in C57BL6/J mice if they were subsequently superinfected. Thus, we determined levels of 23 serum cytokines from C57BL/6J mice 24 hrs after initial inoculation with PBS or UTI89 prior to superinfection. We then superinfected a subset of the mice initially infected with UTI89 (superinfection in Fig. 5) leaving the other mice untouched (UTI89 group). All mice were evaluated with urine titers over 28 d and sacrificed to enumerate bladder titers. We stratified the superinfected mice based on outcome four weeks later as determined by persistent bacteriuria and chronic cystitis. We found elevations of serum KC (Fig. 5A), IL-6 (Fig. 5B), and G-CSF (Fig. 5C) in mice that progressed to chronic cystitis relative to those that resolved infection or were mock-infected with PBS. Therefore, higher levels of these cytokines correlate with chronic cystitis that develops later if mice are superinfected. At the time we obtained serum, the single infection and superinfection groups were identical, and no statistical differences existed among them. These data demonstrate that a subset of C57BL/6J mice respond to an initial infection in a way that results in higher specific serum cytokine levels and primes them to develop chronic cystitis if an additional insult is delivered 24 hpi.

### Response to infection differs between C3H/HeN and C57BL/6J

**Figure 6:**
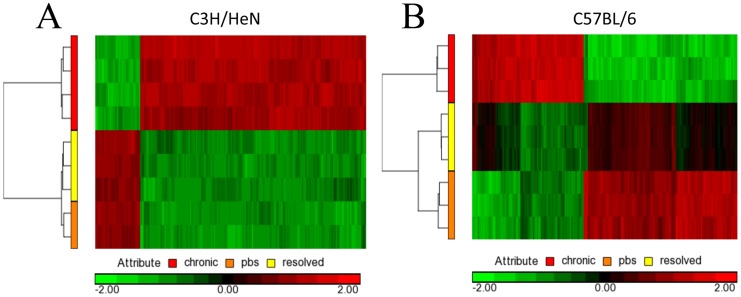
Microarray gene changes for C3H/HeN and C57BL/6J bladders. **A**) C3H/HeN heatmap analysis for mice that resolved infection, experienced chronic cystitis, or were mock infected with PBS. **B**) C57BL/6J heatmap analysis for mice mice that resolved infection, experienced chronic cystitis, or were mock infected with PBS. Depicted is a representative analysis of two biological and three technical replicates.

During chronic cystitis of singly-infected C3H/HeN mice, the bladder epithelium is hyperplastic and normal terminal differentiation of the superficial facet cell layer, including the expression of surface uroplakins, does not occur [37]. In this environment, the bacteria are able to persist extracellularly by an unknown mechanism. To assess this, we conducted scanning electron microscopy analysis on bladder tissue harvested at four wpi and found that bacteria replicate in the presence of ongoing epithelial exfoliation and neutrophil influx in chronic cystitis of both C3H/HeN and C57BL/6J mice (S1A–D Fig.). This analysis supports previous experiments that have shown that during chronic cystitis the majority of bacteria are extracellular, replicating in the urine or adherent to underlying transitional epithelial cells [24], [37]. The mechanism by which bacteria adhere in the absence of uroplakins has not been demonstrated *in vivo*, but *in vitro* studies have shown that FimH binds integrins and other host proteins such as TLR4 [30], [58], [59]. Alternatively additional adhesive factors such as other CUP pili may play a role. Interestingly, during chronic cystitis, neutrophils, which we observed to be actively engulfing bacteria, are insufficient for clearing infection; however, the reason for this is unclear. Mature superficial facet cells could not be discerned at this time point, but were present in mock-infected mice (S1E Fig.). Patients with persistent bacteriuria or rUTI have been reported to have similar histopathology [42]. In order to identify the bladder micro-environment in which UPEC replicate during chronic cystitis, we conducted microarray analysis on RNA extracted from bladders four wpi. C3H/HeN mice were singly-infected and C57BL/6J mice were superinfected to develop chronic cystitis. Mice from each strain inoculated with PBS were used as controls. Depicted in Fig. 6 are the expression profiles relative to the global average with green indicating increased expression and red denoting decreased. C3H/HeN mice experiencing chronic cystitis had a dramatically different expression profile from resolved and mock-infected mice (Fig. 6A). Uroplakins were among the most downregulated genes during chronic cystitis in both mouse models, consistent with the lack of terminally differentiated superficial facet cells (S1 Fig.). Eleven of the 20 (55%) most upregulated genes during chronic cystitis were the same in both mouse strains (S1 Table). The functional categorization revealed that most of the up-regulated genes function in inflammatory response, cytokine release, and ion binding [60]–[62]. Of interest among these genes in both of these mouse models is the inflammasome-related cytokines IL-1. We have shown that UPEC activate the caspase 4 murine homologue, caspase 11, during acute infection in a hemolysin-dependent fashion (Nagamatsu *et. al.* in review). Despite these similarities, interesting differences existed in the ongoing inflammatory response in mice experiencing chronic cystitis (S1 Table). In C57BL/6J mice, the inflammatory response is immunoglobulin- and cytokine-mediated whereas in C3H/HeN mice, we noted a remarkable absence of upregulated immunoglobulin genes. The increased expression of antimicrobial peptides such as RegIIIγ and the calgranulins (s100a8 and s100a9) is interesting because this increased expression is not sufficient to eliminate bacterial replication during chronic cystitis. Interestingly, C3H/HeN mice that were mock infected exhibited a very similar profile to mice that resolve infection (Fig. 6A). Contrary to C3H/HeN mice, C57BL/6J mice that resolved infection differed significantly from either chronic cystitis or mock infected mice, suggesting an element of altered physiology and immunological memory of the infection (Fig. 6B). This information supports research that serially infecting mice that resolve infection makes them less susceptible to recurrent infection [37], [63]. What is interesting here is that the mechanisms by which this occurs may differ between mouse strains, and possibly by extension, women.

## Discussion

We have developed models of bacterial superinfection of the urinary tract, which may provide insight into the connection between recent and frequent sexual intercourse and the susceptibility to the development of chronic UTI [5], [22]. Our results demonstrate that superinfection resulted in increased susceptibility to chronic cystitis in both susceptible and resistant mouse genetic backgrounds, but the time window for priming differed between strains. We have previously shown that chronic cystitis predisposes to severe rUTI upon a subsequent infection weeks to months after clearance of the first infection with antibiotics [37]. Clinically, millions of women take post-coital and prophylactic antibiotics so as not to develop rUTI [64]. Therefore, if clinically applicable, our results detailed here may partially explain why frequent sexual intercourse is such a strong risk factor for UTI. The necessity of prophylactic antibiotics could be obviated if the risk factors and bacterial traits identified here can be altered in the clinical population of women suffering chronic rUTIs.

Frequent sexual intercourse is among the most important risk factors for rUTI in young women [22]. Peri-urethral carriage of the causal strain and sexual intercourse immediately precede the development of a rUTI [5]. Sexual intercourse likely introduces mixed populations of bacteria into the urinary tract, with *E. coli* being the most common [18]. In this environment, UPEC invade bladder tissue and replicate, forming IBCs and bacterial filaments, which have been observed in human urine in 40% of patients suffering acute UTI, 24–48 hours after reported sexual intercourse [21]. These data may provide mechanistic insight as to the frequent clinical observation that recent and frequent sexual intercourse over a brief period of time leads to increased rates of rUTI [23]. Furthermore, elevated levels of serum CSF1, CXCL-1, and CXCL-8 in women with acute UTI were associated with a higher rate of rUTI [45]. Using C3H/HeN and C57BL/6J mice, we have shown that superinfection during the period of acute infection dramatically increases the proportion of mice that experience chronic cystitis with inoculations of 10^7^ UPEC (Fig. 1A and 3A). The bacterial characteristics responsible for frequent recurrences are beginning to be assessed [65]. Hemolysin is expressed by 50% of UPEC isolates, but is more likely to be associated with symptomatic UTI [66]. It is possible that hemolysin-mediated exfoliation and caspase 1/11 activation leads to UTI-associated symptoms. In our studies, we found that an increase in priming for chronic cystitis correlated with the bacterial ability to invade and replicate within the bladder tissue (Fig. 1B–C), and through hemolysin to activate caspase 1/11 leading to IL-1 secretion and bacterial replication (Fig. 1D and 2B). Activation of caspase 1/11 has been shown to contribute to epithelial cell death *in vitro* and exfoliation *in vivo* in C3H/HeN mice, suggesting that caspase-mediated exfoliation may expose the underlying epithelium upon which UPEC replicates during chronic cystitis (Nagamatsu *et. al.* in review). Inhibition of caspase 1/11 protected superinfected mice from chronic cystitis (Fig. 2), suggesting a role for cytokines downstream of caspase activation including IL-1α and IL-1β, identified in our microarray of four-week bladders (Fig. 6; S1 Table). A microarray analysis revealed that in C3H/HeN and C57BL/6J mice, 11/20 of the most upregulated genes during chronic cystitis were the same. Differences between the responses to infection in these mouse strains may result from the dramatic increase in kidney infection or QIR presence in C57BL/6J relative to C3H/HeN mice [37], [40]. Further, this data supports the hypothesis that a muted inflammatory response to UPEC infection is more likely to lead to resolution [26]. Also, our studies suggest that serum biomarkers such as IL-6, KC, and G-CSF may predict a predisposition to rUTI (Fig. 5) [37]. Recently, it was demonstrated that cytokines involved in immune cell chemotaxis and maturation (the human homolog of KC included) during acute UTI enhanced the likelihood of developing rUTI [45].

We have created mouse models that have identified both bacterial and host immune factors that may predispose women to rUTI. Inhibiting caspase-mediated inflammation or downstream effectors may serve to prevent a UTI from becoming a chronic or recurrent UTI. Further work to identify bacterial and host factors that influence the balance between resolution and chronic infection is required to lead to better treatments clinically. The ability of UPEC to invade bladder tissue allows it to transcend stringent bottlenecks during infection [24], [25], [27]. The ability to replicate intracellularly also impacts the ability of a second invading strain to proliferate in the bladder environment (Fig. 1B–C). The molecular basis of bacterial colonization of the bladder during chronic cystitis is an area of active investigation. Previously, it has been shown that mannosides are effective in treating chronic cystitis arguing that FimH-mediated binding plays an important role [67]. It has recently been demonstrated that FimH variation outside of the binding pocket affects protein conformation and pathogenicity of UPEC [48]. This variation may impact bacterial adherence and replication during chronic cystitis. Furthermore, because invasion and intracellular replication appear to influence the likelihood to develop chronic cystitis, treatments with soluble compounds such as mannosides that block the ability of UPEC to invade the tissue or compounds that might alter FimH conformation hold promise as effective means to prevent or treat rUTI [67]–[70]. These analyses may allow us to identify high-risk patients for more aggressive therapy and/or anti-virulence compounds to limit this troubling disease.

## Materials and Methods

### Bacterial strains

All WT bacterial strains utilized were derivatives of UTI89, including tagged, isogenic UTI89 isolates, kanamycin resistant UTI89 *att_HK022_*::COM-GFP, kanamycin resistant UTI89 with re-integrated UTI89 FimH, spectinomycin resistant UTI89 *att_λ_::PSSH10-1*, and chloramphenicol resistant UTI89 [24], [47], [71]. FimH mutant strains, *ΔompA*, *Δkps*, *ΔhlyA*, *ΔcpxR* were all previously published [47], [51], [52](Nagamatsu *et al.* in review).

### Mouse infections

Bacteria for infection were prepared as previously described [72]. Six to seven week old female C3H/HeN (Harlan) or C57BL/6J (Jackson) were transurethrally infected with a 50 µL suspension containing 5×10^6^–2×10^7^ CFU of UTI89 or relevant mutant in PBS under 3% isofluorane. Protamine Sulfate (Sigma) was dissolved in PBS and caspase 1/11 inhibitor Ac-YVAD-CMK (BACHEM) was dissolved in DMSO and transurethrally inoculated into the bladder. At indicated timepoints after infection, mice were anesthetized and infected again. Venous blood was obtained at 24 hpi, just prior to re-infection, by submandibular puncture and centrifuged at max speed at 4°C in Microtainer serum separation tubes (BD) and stored at −20°C until use. Cytokine expression was measured using the Bio-Plex multiplex cytokine Group I bead kit array (Bio-Rad), which measures 23 cytokines. Urine was obtained by gentle suprapubic pressure and serially diluted and plated on appropriate antibiotic plates. Mice were sacrificed by cervical dislocation under isofluorane anesthesia, and their organs were aseptically removed. Chronic cystitis was determined if animals had urine titers>10^4^ CFU/mL at 1, 7, 14, 21 dpi and bladder titers>10^4^ CFU at sacrifice at 28 dpi [37]. Animals that resolved infection and had a recurrence or had resolved the infection with reservoir titers>10^4^ CFU were marked in red and considered to have resolved the chronic infection. Organ titers shown are the total bacterial burden.

### Ethics statement

The Washington University Animal Studies Committee approved all mouse infections and procedures as part of protocol number 20120216, which was approved 01/11/2013 and expires 01/11/2016. Overall care of the animals was consistent with *The guide for the Care and Use of Laboratory Animals* from the National Research Council and the USDA *Animal Care Resource Guide*. Euthanasia procedures are consistent with the “AVMA guidelines for the Euthanasia of Animals 2013 edition.”

### Microarray experiments

C3H/HeN or C57BL/6J mice were infected as discussed above. After 28 days, animals that had developed chronic cystitis, resolved the infection, or aged matched PBS controls were sacrificed for RNA isolation. Upon sacrifice, 5 bladders from each condition were immediately pooled and homogenized in Trizol for RNA isolation according to the manufacture's suggested protocol. DNase treatment was performed to remove any contaminating DNA before submission to the Genome Technology Access Center for sample processing and hybridization on Affymetrix Mouse Gene 1.0 chips in triplicate. Data was analyzed using the Partek Genomics Suite. Gene lists were compiled using fdr-ANOVA analysis with a significance cut off of p<0.001. Experiments were repeated twice with a representative analysis shown. Microarray data are available in the ArrayExpress database (www.ebi.ac.uk/arrayexpress) under accession number E-MTAB-2930.

### Scanning electron microscopy

Mice were infected as described above. Bladders were aseptically harvested, bisected, and splayed. Bladders were fixed in 2.0% glutaraldehyde in 0.1M sodium phosphate buffer overnight. Bladders were then washed three times with 0.1M sodium phosphate buffer and de-ionized water before being fixed in 1.0% osmium tetroxide. Bladders were washed and then critical point drying was performed with absolute ethanol and liquid carbon dioxide. Sputter coating was performed with gold-palladium using a Tousimis Samsputter-2a. Images were obtained on a Hitachi S-2600H operated at 20 kV accelerating voltage.

### Statistical analysis

Datapoints below the limit of detection (LOD) were set to the LOD for graphical representation and statistical analysis. For cytokine data, values out of the range of the instrument were not included for analysis. Fisher's exact test was utilized to determine differences between groups for rates of chronic cystitis. One-way ANOVA was utilized to determine whether any cytokine differences were apparent and pairwise assessment of median values was determined by Mann-Whitney test. Unless otherwise indicated, p<0.05 was considered significant. Analyses were performed in Graphpad Prism 5.0.

## Supporting Information

S1 Fig **Bacteria replicate on the bladder surface during chronic cystitis.** Bladders of C3H/HeN and C57BL6 mice were splayed four wpi and fixed in glutaraldehyde. **A–B**) Chronic C3H/HeN bladders. **C–D**) Chronic C57BL/6J bladders. **E**) Mock infected C57BL/6J bladder shown for comparison.
(TIF)

S1 Table **Genes with highest fold change of chronic cystitis versus resolved.**
(PDF)
