# Supplementary figures and images for: Uropathogenic Escherichia coli Superinfection Enhances the Severity of Mouse Bladder Infection
Source: PLoS Pathog. 2015 Jan 8;11(1):e1004599. doi: 10.1371/journal.ppat.1004599 (PMC4287616; doi:10.1371/journal.ppat.1004599)

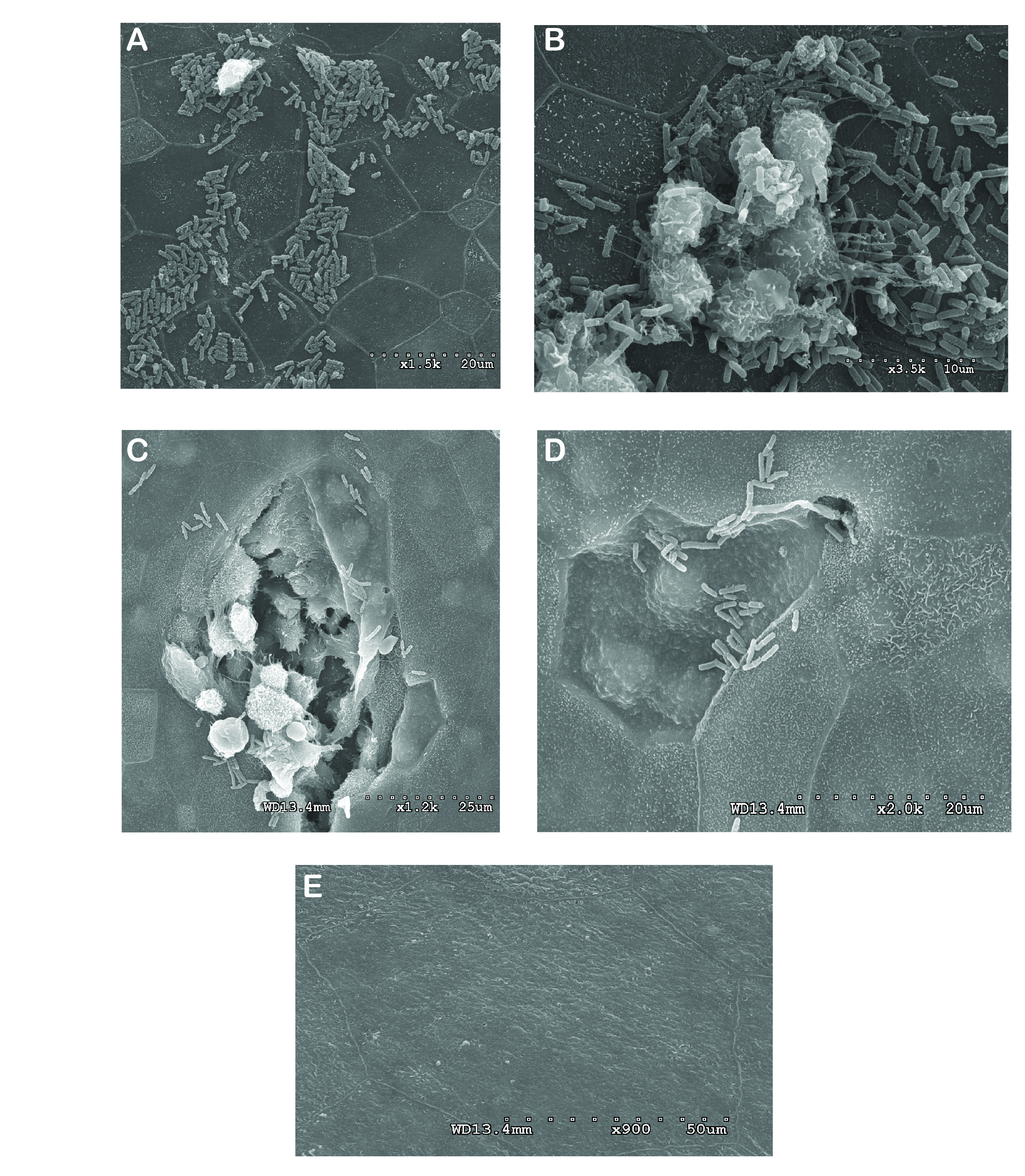

Supplement: S1 Fig — Bacteria replicate on the bladder surface during chronic cystitis. Bladders of C3H/HeN and C57BL6 mice were splayed four wpi and fixed in glutaraldehyde. A–B) Chronic C3H/HeN bladders. C–D) Chronic C57BL/6J bladders. E) Mock infected C57BL/6J bladder shown for comparison. (TIF) [file ppat.1004599.s001.tif]
