# Supplementary material for: Uropathogenic Escherichia coli Superinfection Enhances the Severity of Mouse Bladder Infection
Source: PLoS Pathog. 2015 Jan 8;11(1):e1004599. doi: 10.1371/journal.ppat.1004599 (PMC4287616; doi:10.1371/journal.ppat.1004599)
Supplement: S1 Table — Genes with highest fold change of chronic cystitis versus resolved. (PDF) [file ppat.1004599.s002.pdf]

C57BL6			C3H/HeN		
Gene name	Fold change (chronic v resolved)	Functional Annotation	Gene name	Fold change (chronic v resolved)	Functional Annotation
**Ubd**	**119.405**	**Proteosome**	BC100530	144.401	Unknown
**Saa3**	**112.203**	**Inflammatory Response**	**S100a8**	**88.7327**	**Cytokine/Ion Binding**
**S100a8**	**85.4193**	**Cytokine/ Ion Binding**	**Mmp7**	**87.0008**	**Ion Binding**
**Mmp7**	**77.6183**	**Ion Binding**	**Ubd**	**77.6292**	**Proteosome**
**RegIIIγ**	**66.2146**	**Inflammatory Response**	**S100a9**	**74.4965**	**Cytokine/ Ion Binding**
**S100a9**	**44.9876**	**Cytokine/ Ion Binding**	**RegIIIγ**	**62.1077**	**Inflammatory Response**
Igk-V19-14	31.1745	Immunoglobulin	**Cxcl2**	**39.3707**	**Cytokine**
Igk-V28	27.7003	Immunoglobulin	Il1b	38.9361	Cytokine
**Fam3b**	**25.7301**	**Cytokine**	**Sprr2f**	**38.7662**	**Tissue Remodeling**
Plekhs1	24.3332	Tissue Remodeling	**Saa3**	**36.8235**	**Inflammatory Response**
Duox2	23.492	Ion Binding	Cxcl5	34.2451	Cytokine
Gm5571	22.2598	Immunoglobulin	**Il1a**	**33.0689**	**Cytokine**
**Sprr2f**	**22.0386**	**Tissue Remodeling**	Gabrp	31.6717	Ion Binding
AA467197	21.9697		Sprr2d	31.4934	Tissue Remodeling
Cxcl9	21.46	Cytokine	**Fam3b**	**29.3965**	**Cytokine**
**Cxcl2**	**17.698**	**Cytokine**	Cxcr2	27.6748	Cytokine
Pigr	17.1008	Immunoglobulin	**Tmprss11g**	**26.263**	**Tissue Remodeling**
**Tmprss11g**	**15.7865**	**Tissue Remodeling**	Lcn2	25.9349	Ion Binding
Nos2	15.644	Inflammatory Response	Il1f9	24.0818	Cytokine
**Il1a**	**15.0219**	**Cytokine**	Sprr2g	23.4676	Tissue Remodeling
